# A Gq Biased Small Molecule Active at the TSH Receptor

**DOI:** 10.3389/fendo.2020.00372

**Published:** 2020-06-26

**Authors:** Rauf Latif, Syed A. Morshed, Risheng Ma, Bengu Tokat, Mihaly Mezei, Terry F. Davies

**Affiliations:** ^1^Thyroid Research Unit, Icahn School of Medicine at Mount Sinai, New York, NY, United States; ^2^James J. Peters VA Medical Center, New York, NY, United States; ^3^Department of Pharmacological Sciences, Icahn School of Medicine at Mount Sinai, New York, NY, United States

**Keywords:** TSH, GPCR, gprotein, proliferation, agonist

## Abstract

G protein coupled receptors (GPCRs) can lead to G protein and non-G protein initiated signals. By virtue of its structural property, the TSH receptor (TSHR) has a unique ability to engage different G proteins making it highly amenable to selective signaling. In this study, we describe the identification and characterization of a novel small molecule agonist to the TSHR which induces primary engagement with G_αq/11_. To identify allosteric modulators inducing selective signaling of the TSHR we used a transcriptional-based luciferase assay system with CHO-TSHR cells stably expressing response elements (CRE, NFAT, SRF, or SRE) that were capable of measuring signals emanating from the coupling of G_α*s*_, G_α*q*/11_, G_βγ_, and G_α12/13_, respectively. Using this system, TSH activated G_α*s*_, G_α*q*/11_, and G_α12/13_ but not G_βγ_. On screening a library of 50K molecules at 0.1,1.0 and 10 μM, we identified a novel G_q/11_ agonist (named MSq1) which activated G_q/11_ mediated NFAT-luciferase >4 fold above baseline and had an EC_50_= 8.3 × 10^−9^ M with only minor induction of G_αs_ and cAMP. Furthermore, MSq1 is chemically and structurally distinct from any of the previously reported TSHR agonist molecules. Docking studies using a TSHR transmembrane domain (TMD) model indicated that MSq1 had contact points on helices H1, H2, H3, and H7 in the hydrophobic pocket of the TMD and also with the extracellular loops. On co-treatment with TSH, MSq1 suppressed TSH-induced proliferation of thyrocytes in a dose-dependent manner but lacked the intrinsic ability to influence basal thyrocyte proliferation. This unexpected inhibitory property of MSq1 could be blocked in the presence of a PKC inhibitor resulting in derepressing TSH induced protein kinase A (PKA) signals and resulting in the induction of proliferation. Thus, the inhibitory effect of MSq1 on proliferation resided in its capacity to overtly activate protein kinase C (PKC) which in turn suppressed the proliferative signal induced by activation of the predomiant cAMP-PKA pathway of the TSHR. Treatment of rat thyroid cells (FRTL5) with MSq1 did not show any upregulation of gene expression of the key thyroid specific markers such as thyroglobulin(Tg), thyroid peroxidase (Tpo), sodium iodide symporter (Nis), and the TSH receptor (Tshr) further suggesting lack of involvement of MSq1 and G_αq/11_ activation with cellular differentation. In summary, we identified and characterized a novel G_αq/11_ agonist molecule acting at the TSHR and which showed a marked anti-proliferative ability. Hence, Gq biased activation of the TSHR is capable of ameliorating the proliferative signals from its orthosteric ligand and may offer a therapeutic option for thyroid growth modulation.

## Introduction

Traditionally GPCR drug development has focused on conventional agonists and antagonists that are known to act as “on-off” switches. However, there is growing appreciation that GPCRs can mediate their physiologically relevant effects through selective signaling due to subtle structural changes and engagement of G protein and non-G protein effectors. Selective signaling can be driven by endogenous ligands, synthetic peptides or small molecules, which bind to the orthosteric or allosteric site(s) and in turn bias the downstream signal. The TSHR which is made up of a large glycosylated ectodomain and seven transmembrane helices which are connected by extracellular and intracellular loops (1) is structurally poised as a candidate for allosteric modulation with its ability to engage all four classes of G proteins (2). Studies using both modeling and mutational analysis of the TSHR have indicated the structural determinants of the G protein coupling to the receptor (3, 4). However, it is not yet fully clear as to what preferential order these different G proteins are engaged by the TSHR during activation nor the exact intra- and -inter molecular interactions leading to coupling of the different G proteins by TSH or TSHR antibodies. However, crystallization of the partial ectodomain with stimulating and blocking autoantibodies (5–7) together with studies of the molecular rearrangement of the TSHR ectodomain and hinge regions has given some recent insight into the possible mechanism(s) of this activation (8, 9).

Small molecules can bind to the allosteric sites on the TSHR TMD and ectodomain (10, 11) and are excellent tools to gain insight into the potential for TSHR selective signaling. Their unique ability to readily permeate the cell membrane and interact with specific residues within the transmembrane helices can induce subtle conformational changes (12–14). In recent years there has been rapid development of small molecules, both agonists (15–17) and antagonists (16, 18–20) against the TSHR as part of a search for novel therapeutic agents. These various small molecule ligands induce the Gα_*s*_ pathway of the TSHR and the possibility of selective Gα_q/11_ activation by a small molecule has not been explored. However, studies have indicated that such selectivity in signaling can be established in GPCRs and not only by different receptor subtypes (21, 22) but also via pathway bias suggesting ligand selectivity can be a potential source of a defined pharmacology for small molecules (23, 24).

In this report, we describe the identification and *in vitro* characterization of a novel small molecule that activates the TSHR by preferentially initiating Gα_q/11_ signaling and then examined its biological consequences on thyrocyte proliferation and gene expression.

## Materials and Methods

### Establishing Double Transfected CHO-TSHR Cell Lines

In order to identify the signaling through the four major classes of G-proteins (G_α*s*_,G_α*q*/11_ and G_α12/13_ and G_βγ/*i*_) by the TSHR, we generated double transfected CHO-TSHR stable lines containing CRE, NFAT, SRF, or SRE response elements (RE) tagged to a modified form of luciferase reporter. These double transfected stable clones were established by selecting the cells with hygromycin (800 ug/ml) and 500 ug/ml of G418 (neomycin sulfate). Following initial screening and validation, these stable cell lines were maintained in Ham's F-12 medium with 10% fetal bovine serum (FBS), 100 units of penicillin and streptomycin with 200 ug/ml of hygromycin and G418 to maintain the selection pressure in these co-transfected cells. Using the individually co-transfected stable lines containing the respective response elements, we screened a 50K chemical library at 0.1,1 and 10 μM against CRE, NFAT, SRF, and SRE cells in a 384 well format following the protocol described previously (17).

### Treatment and Lysate Preparation

For downstream signaling studies, low passage number of FRTL5 cells were cultured in 60 mm dishes using Hams F12 medium with 5% calf serum to which 1X 6H (6 hormone mixture) was added as previously described (25). Once the cells reached 60–80% confluence, cells were washed twice with plain medium and then cultured further in Ham F12 medium containing only 5H hormone (-TSH) for 72 hrs. Following this the cells were washed twice with plain F12 medium and incubated for another 48 h in Ham's F12 medium containing 0.3% BSA (basal medium). These cells were then either stimulated with increasing dose of MSq1, TSH or MS438 or combination of TSH plus MSq1 or TSH+MSq1+ PKC inhibitor at 2 μM (G06883) as per the experimental details described under figure legends for 48 h at 37C. Lysates from these treated cells were prepared using 1X Novagen phosphosafe extraction buffer as per the manufacturer's instructions and total protein in the lysate estimated by Bradford (26). Further the proteins were resolved on 4–15% SDS-PAGE and transferred to PVDF membranes by wet transfer and classic immunblotting performed for detection of phospho protein after blocking membranes with 2% BSA for 2 h at RT or subjected to protein quantification using simple western system by the WES machine for immunoblotting and detection (ProteinSimple, Santa Clara, CA, USA).

### Immunoblotting and Detection

In the present study, we quantitated the absolute response to PKC and PKA in the lysate prepared from the treated cells as descried above. pPKC was detected by classical immunoblotting procedure described earlier (27) using commercially obtained primary antibodies to pPKC βII ser660, Anti-rabbit HRP (1:20,000) in 1X tris-borate saline with tween 20 0.5% (TBST) was used as detection antibody and the immunoblots developed with ECL. Quantitation of pPKA was carried out using the protein simple WES system after titrating the primary and secondary antibodies against different concentrations of the samples. Briefly, the WES protocol is as described here, first, a 0.2 μg of lysate was mixed with master mix to achieve a final concentration of 1X sample buffer in the presence of fluorescent molecular weight marker and 40 mM dithiothreitol, the samples were denatured at 95°C for 5 min. Target proteins were immuno-probed with primary antibody pPKA (thr197) followed by HRP-conjugated secondary antibodies. All antibodies were diluted using an antibody diluent at a 1:100 or 1:200. Detection of ERK 44-kDa protein in the lysate using anti ERK served as a positive run control in addition to the biotinylated ladder for size estimation. β-actin was used as the loading control. Digital images of the signal were analyzed with Compass software (ProteinSimple), and the quantified data of the detected proteins with the correct molecular weight is reported as signal/noise ratio derived from average signal intensity exposures.

### Proliferation Measured by Alamar Blue

Proliferation of FRTL5 cells was measured using Alamar Blue, which monitors the reducing environment of the living cell. The active ingredient is resazurin, which is a stable, nontoxic and permeable compound, which accepts electrons and changes from the oxidized, non-fluorescent, blue state to the reduced, fluorescent, pink state. These studies were carried out on FRTL5 grown on black clear bottom 96 well plates. Cells in the log phase were harvested by trypsin and seeded as 30 × 10^3^ cells/well and allowed to adhere to the bottom of plate in complete HamF12 medium by incubating the cells with 6H overnight at 37°C. Following a 24 to 36 hrs incubation, the cells were culturally prepared by removing TSH for 3 days prior to induction of proliferation as described earlier. The cells were then exposed to MSq1, TSH or combination of both with and without the PKC inhibitor as per the experiment described under figure legends. For determining the effect of a small molecule or TSH on cell growth, we had stimulated vs. unstimulated cells. Following 48 h of treatment, Alamar Blue was aseptically added to each well in an amount equal to 10% of the volume in the wells. Cells with Alamar Blue were further incubated at 37°C for another 5 h prior to reading the plates. Proliferation was assessed by measuring fluorescence intensity of the reduced dye at 540/580 nm. Wells with media plus dye only was used as the background control. Log change between untreated over that of treated groups was deduced from the fluorescent intensities obtained after background subtraction.

### Docking and Contact sites

Docking of the lead MSq1 molecules was performed on a homology model of the TSHR-TMD based on rhodopsin (PDB:1F88). This template was chosen because of the low RMSD values between the backbone of the TM helices of the TSHR model and that of the rhodopsin x-ray crystal structure (14) and fits the experimental parameters that we have previously described (15). The initial homology model of rhodopsin TMD was obtained from the Uniprot server (http://www.uniprot.org). The conformations of the extracellular loops were constructed with a Monte Carlo method (16). The 3D geometries of the docked ligands were generated with MarvinSketch (http://www.chemaxon.com). Multiple docking was carried out using the programs Glide, Autodock-4 and Autodock-Vina. The docking results were analyzed using Dockres and other supporting script tools (17). In particular, Dockres extracts the coordinates of the docked poses from the docking log file and identifies contacts between the ligand and target as pairs of mutually proximal atoms and hydrogen bonds (if any) as X…H-X' where X and X' are polar atoms (one on the ligand and the other on the target) with X…H distance within threshold and X…H-X angle is greater than 120 deg.

### IP-One Assay

In principle PLC is the main intracellular effector enzyme of G_αq/11_-coupled GPCRs. PLC hydrolyzes PIP_2_ into IP_3_ and DAG. The intracellular second messenger IP_3_ is rapidly degraded by phosphatases and recycled back via inositol into cell membrane PIP_2_. Thus, for measuring G_αq/11_ activation by MSq1 in CHOTSHR cells we used the Cisbio IP-One Gq kit which is a competitive immunoassay intended to measure myo-inositol-1phosphate (IP1) accumulation in cells. The inositol phosphate accumulation assay utilizes the ability of lithium to inhibit the breakdown of inositol monophosphates and detects this accumulated IP1 by HTRF® technology. In the assay native IP1 produced by cells or unlabeled IP1 (standard curve) compete with d2-labeled IP1 (acceptor) for binding to anti-IP1-Cryptate (donor). The specific signal (i.e., energy transfer) is inversely proportional to the concentration of IP1 in the standard or sample. 50 × 10^3^ CHOTSHR cells per well were seeded in 96 well black plates in complete Hams F12 medium and incubated overnight at 37°C. The adherent cells were gently washed once with warm plain medium with low serum (2%) and the cells were treated with increasing doses of TSH (μU) or MSq1 (μM) in stimulation buffer containing 50 mM of lithium chloride. At the end of 2 h incubation the cells were lysed using the lysis buffer provided and treated with detection antibodies as per manufacture's instructions and run along with the standards provided in the kit. The measurement of acceptor (665 nm) to donor (620 nM) emission was obtained using the microplate plate reader ClarioStar and ratio calculated and interpolated to standard curves to calculate the values of the unknown samples.

### TSHR Expression by Flow-Cytometry

ML-1 and FT236, two follicular cancer lines, were grown in DMEM high glucose with 10 % FBS, 200 mM glutamine, 1x sodium pyruvate 1X Minimum essential medium with 100 units of penicillin and streptomycin. The cells were detached from the plate non-enzymatically using 1 mM EGTA/EDTA and washed twice with 1X PBS, filtered using 75 micron filter and total cells counted. 0.5 × 10^6^ cells/tube were suspended in 100 ul of FACS staining buffer (1X PBS with 0.2% sodium azide and 2% FBS) with anti TSHR mAb RSR1 mouse Mab (0.1 μg/ml) and incubated for 1 h at room temperature. Following 2x wash with FACS buffer (1XPBS with 0.02% sodium azide) and the bound TSHR receptor antibodies were detected using anti-mouse antibody Fab' phycoerthrin (PE) labeled secondary antibody at 1:200. Unstained cells, isotype antibody or secondary antibody alone were used as controls in the assay. The results were expressed as the percentage positive cells detected in the test samples compared to the controls by the vertical gates assigned based on the controls.

### Gene Expression

For gene expression analysis, total RNA was extracted using a RNeasy kit and was treated with ribonuclease-free deoxyribonuclease. Five micrograms of total RNA were reverse transcribed into cDNA using the SuperScript III system. All Q-PCRs was performed using the Step OnePlus Real-time PCR system (Applied Biosystems, Foster City, CA). The reactions were established with 10 μL of SYBR Green master mix (Applied Biosystems, Foster City, CA), 0.4 μl (2 μM) of sense/anti-sense gene-specific primers, 2 μl of cDNA and DEPC-treated water to a final volume of 20 μl. The PCR reaction mix was denatured at 95°C for 60 s before the first PCR cycle. The thermal cycle profile was used is as follows: denaturizing for 30 s at 95°C; annealing for 30 s at 57–60°C (dependent on primers); and extension for 60 s at 72°C. A total of 40 PCR cycles were used. For each target gene, the relative gene expression was normalized to that of the glyceraldehyde-3-phosphate dehydrogenase (GAPDH) housekeeping gene. Data presented as fold change in relative gene expression are from two independent experiments in which all sample sets was analyzed in triplicate.

### Statistical Analyses

All curve fitting and P value calculations (one-way ANOVA) were carried using GraphPad Prism 5 software. All assays were performed at least 2 or 3 times as indicated. In case of immunoblot one representative experiment is shown.

## Results

### Identification of a Unique Gq Activator

**Figure 1:**
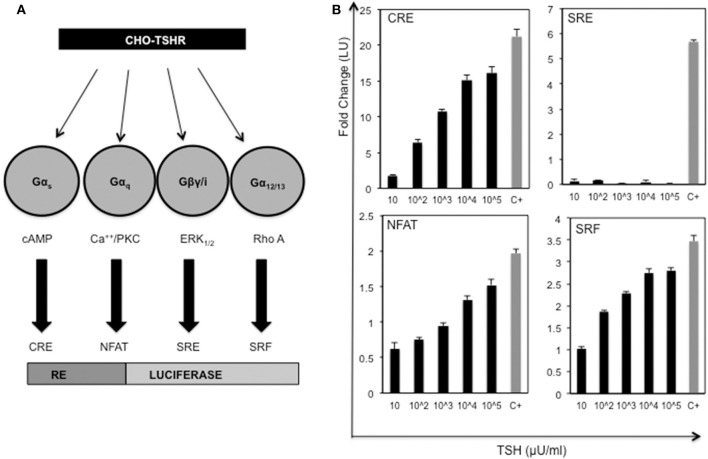
Activation of the TSH receptor and G proteins. **(A)** Schematic representation of double transfected CHO-TSHR cells generated to study activation of the different G proteins. Shown are the 4 luciferase tagged response elements (REs) that are capable of measuring the activation of the respective second messengers used by the G proteins. **(B)** The bar graph panels represent the dose-responses with TSH (10 μU to 10^5^ μU/ml) and the respective positive controls used with each of the double transfected stable cells. The change in activity is represented as fold change of luciferase units (LU) on the Y-axis. The gray bars marked with C+ in the x -axis are the positive controls for the each of the response elements. The positive controls used were as follows: CRE - forskolin 5 uM, NFAT- ionomycin 100 μM, SRF- 20% serum + PMA 10 ng and SRE−20% serum. The data represented here are from 3 separate experiments. Note that all the data shown here are baseline subtracted.

In order to identify allosteric ligands that can activate different G proteins of the TSHR we first developed a series of CHO-TSHR cells that were transfected with different response elements tagged to luciferase that can specifically identify the activation of specific G proteins as indicated schematically in Figure 1A. The activation of these different response elements was validated using bovine TSH as indicated in Figure 1B. This analysis clearly indicated that TSH was capable of activating Gα_s_, Gα_q/11_, and Gα_12/13_ in a dose-dependent manner. No activation was observed of Gβγ in this system. The respective positive controls used for each of the response elements are indicated and explained in the figure legends.

**Figure 2:**
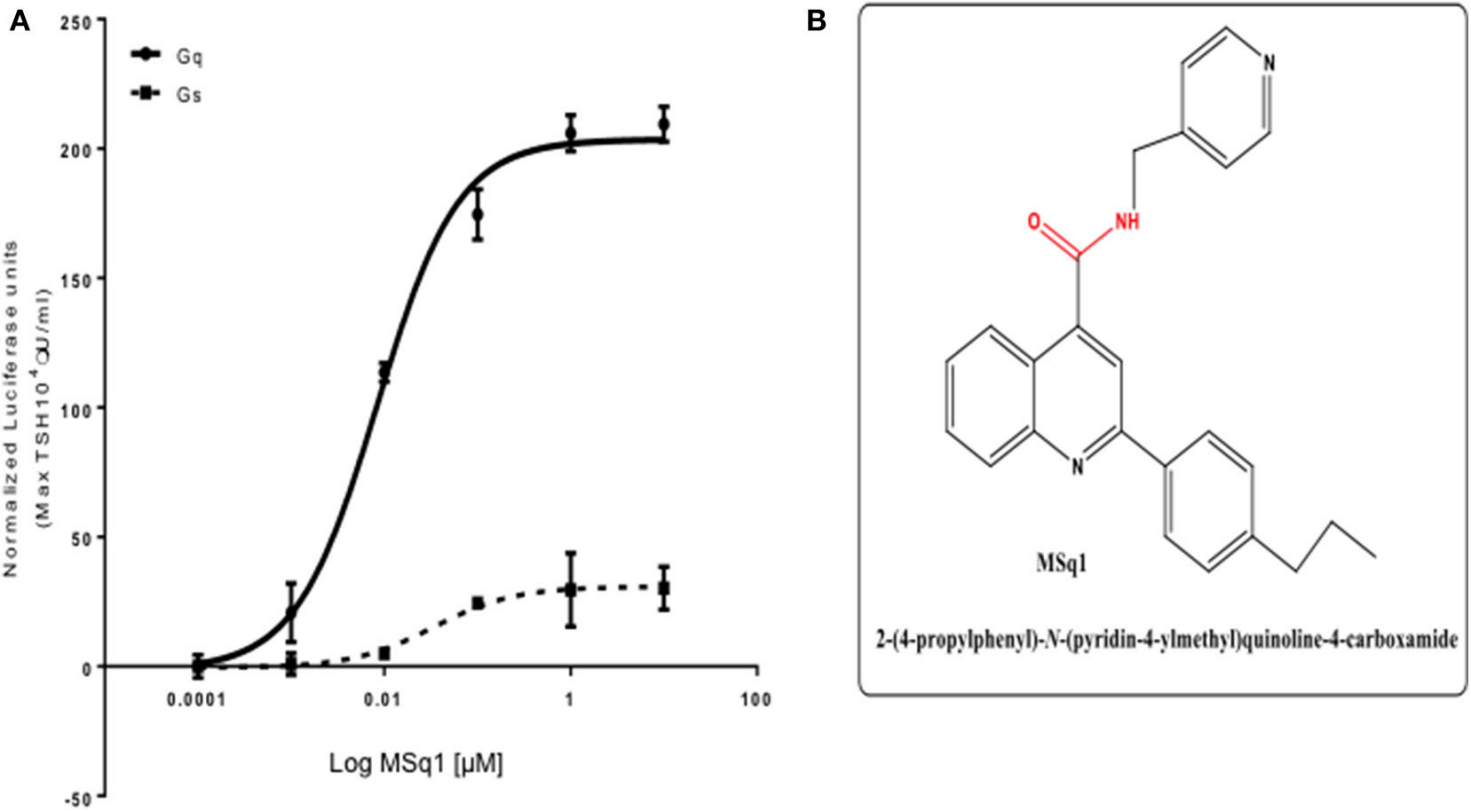
**(A)** Potency of selected Gα_q/11_ agonist MSq1. Dose-response relationship of MSq1 against the NFAT and CRE response element containing cells. A 4-fold increase of NFAT luciferase was observed with MSq1 compared to a small increase in CRE luciferase showing that MSq1 is biased toward Gα_q/11_ signaling. **(B)** Molecular structure of MSq1. Chemical structure and SMILE of Msq1. The blue represents the nitrogen atoms and red corresponds to the oxygen atoms in a carbon backbone. Chemically it is 2-(4-propylphenyl)-N-(4-pyridinylmethyl)-4-quinolinecarboxamide. Name:C1(C2=CC=CC=C2N=C(C2C=CC(=CC=2) CCC) C=1) C(=O)NCC1C=CN=CC=1.

Screening a 50K library at 0.1,1 and 10 μM against this panel of stable CHO TSHR luciferase cells allowed us to identify a small molecule, which preferentially activated CHO-TSHR-NFAT luciferase cells. Further examination of this Gq activator (named MSq1) against CHOTSHR-NFAT, which couples G_αq/11_, and CHOTSHR-CRE, which measures activation via Gα_s_, in a dose-dependent manner (Figure 2A) showed MSq1 to be a potent activator of Gq with an EC_50_ = 8.3 × 10^−9^M after normalizing the data to max TSH (10^4^μU/ml). MSq1 had only minor activation toward Gs thus making this molecule a preferential Gq activator. Structurally this molecule differed from any of the known agonist or antagonist small molecules (Figure 2B). Control studies with MSq1 measuring its influence on activation in normal CHO cells (without a TSHR) but transfected with either NFAT luciferase or CRE luciferase at 10 μM showed no activation of luciferase (Figure S1). We have shown that MSq1 is incapable of activating either G_βγ_ or G_12/13_ using the luciferase system further confirming that this is a G_*q*/11_ biased novel small molecule (Figure S2).

### Binding Sites of MSq1 by Docking Studies

**Figure 3:**
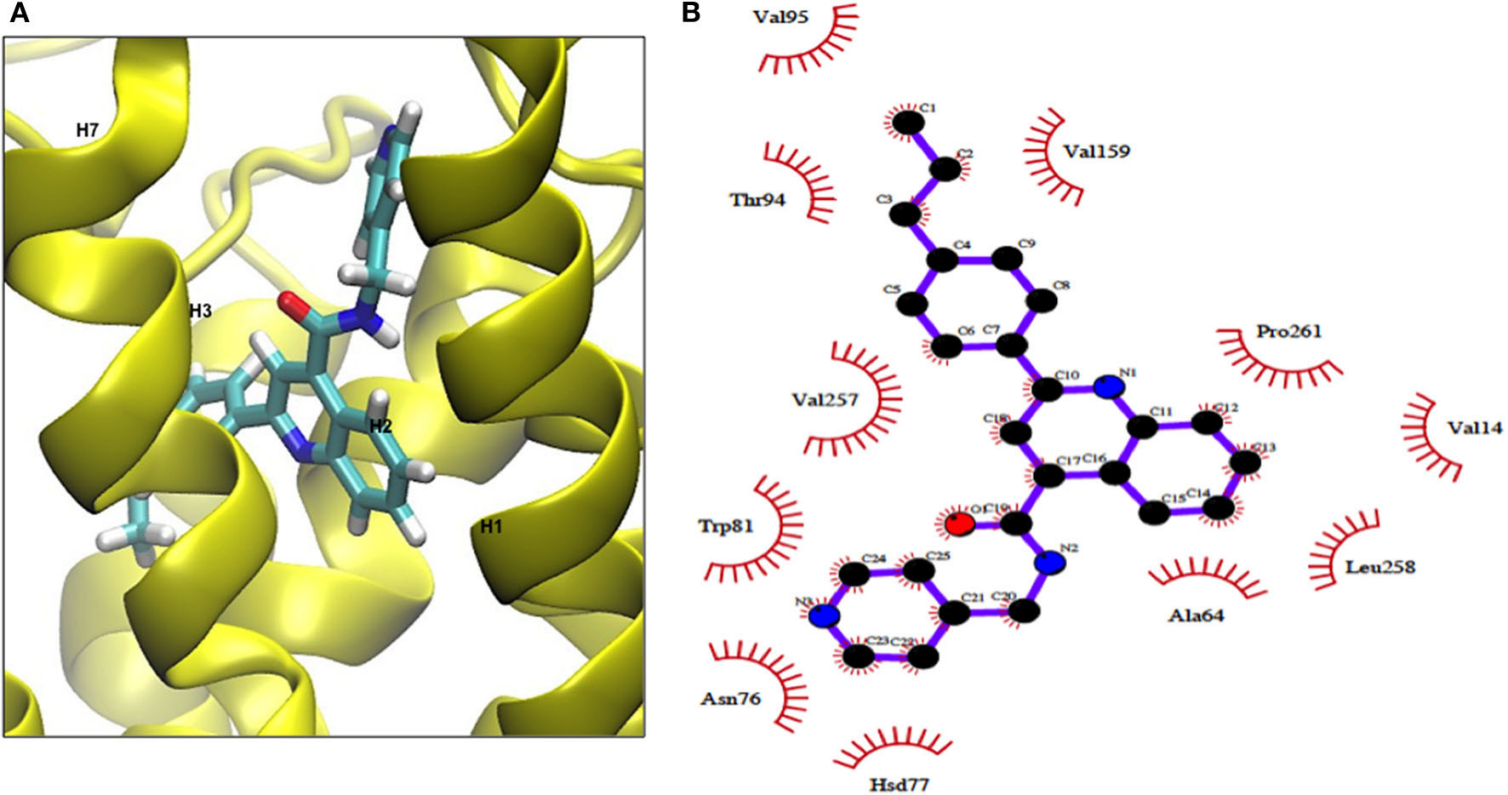
**(A)** Binding of MSq1 molecule to the TSHR TMD. A homology model of the TSHR transmembrane domain, previously described (28), was used as the template for docking studies. Analysis of the Autodock results as detailed in Materials and Methods indicated that MSq1, like other small molecules, docks into a hydrophobic pocket of the TSHR TMD and in this case makes contact with residues in helices H1, H2, H3, H6, and H7 and the extracellular loops 2,3 and 4,5. **(B)** The TSHR TMD and its contact sites with MSq1. On extracting co-ordinates of the docked poses using Dockres, the program showed contact resides against the TSHR TMD (red semi asterisks) assessed by the criteria of ≤ 4Å as indicated in this diagram. Furthermore, these contact residues in the TSHR TMD and their location within the TMD residues are indicated along with the contacts for MS438 in Table 1 for comparison.

We examined the binding sites of this Gq activator by *in-silico* docking using the structure of the TSHR TMD region developed by homology modeling and based on the rhodopsin crystal structure (as detailed in Methods). Using the top scoring docking poses generated by Autodock-4 and the criterion of ≤ 4Å, the putative contact points of MSq1 within the TSHR TMD were deduced. Like most allosteric small molecules against the TSHR, the MSq1 sites were nestled in the “hydrophobic pocket” formed by the different helices within the TSHR TMD (Figure 3A). Further analysis indicated that MSq1 made major contact points on the TSHR TMD helices H1, H2, H3, and H7 within the hydrophobic pocket and the extracellular loops including L2-3 & L4-5 (Figure 3B). When these contact residues were compared to our Gα_s_ agonist MS438 some overlapping, and some unique residues could be observed as shown in Table 1 which lists the top-scoring Glide, Autodock-4 and Autodock-Vina poses for both MS438 and MSq1.

**Table 1:** TSHR residues on the TMD that MSq1 and MS438 contact.

			**MS438**			**MSq1**		
**TSHR residue**	**Residue No #**	**Ballest #**	**rG(l-P)**	**rA(L-P)**	**rV(l-p)**	**rG(L-P)**	**rA(L-P)**	**rV(L-P)**
LEU	10	1.35		3.1	3.6		3.1	
VAL	14	1.39			3.2			3.4
VAL	17	1.42			3.4			
LEU	60	2.57		3.2	3.2			3.2
LEU	61	2.58		3.2	3.1			
ALA	64	2.57	2.9	2.8	3.5	2.9	2.8	
ASN	76	L (2-3)	3.1	2.8	3.6	2.9		
TRP	81	L (2-3)			3.8	3.1		
CYS	87	3.25	3.6			3.6		
ALA	90	3.28	4.0		3.7	4.0		
GLY	91	3.29	3.0		3.6			3.6
THR	94	3.32		2.8	2.7	3.8	3.5	3.7
VAL	95	3.33		3.1	3.6		3.1	3.6
SER	98	3.36		3.3	3.4		3.4	3.4
GLU	99	3.37			3.7			
LYS	158	L (4-5)		3.2				
VAL	159	L (4-5)		3.4	3.5	3.7		4.1
ILE	233	6.51		3.2	3.4			3.4
LYS	253	7.42		3.3	3.8		3.3	
ILE	254	7.43		3.3				
VAL	257	7.46	3.7	2.7	3.6	3.2	3.3	3.4
LEU	258	7.47		3.1	3.2		3.5	3.4
TYR	260	7.49		3.2			3.5	
PRO	261	7.50	3.1	3.0	3.6	3.1		3.7

### Downstream Signaling of the Gq Activator

**Figure 4:**
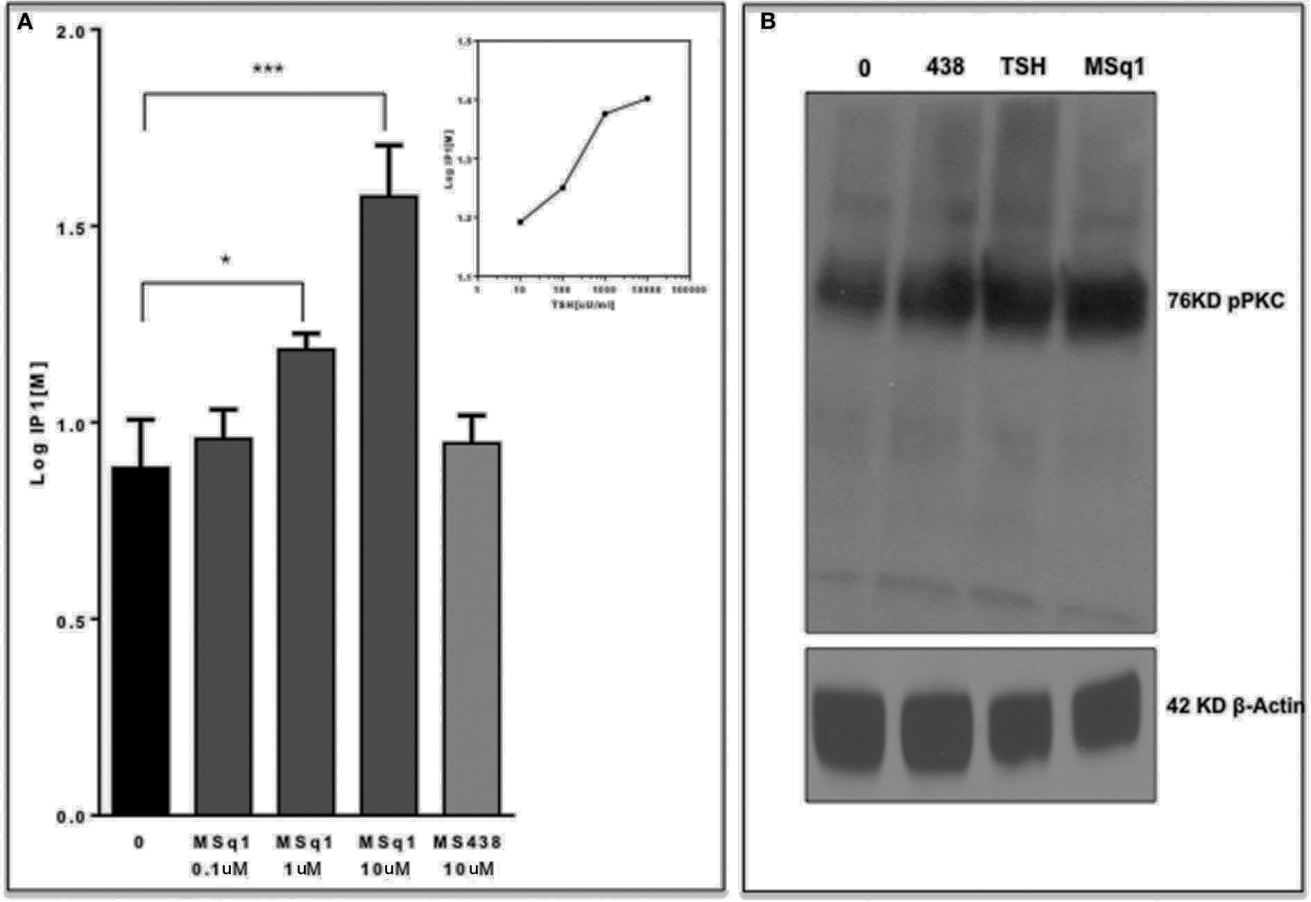
Gq signaling by MSq1. **(A)** Since Gq activation is known to result in an IP1 increase via PLC-β activation, we measured IP activation in CHO-TSHR cells with MSq1 at 0.1 and 10 μM. As indicated here MSq1 showed a significant increase (*P* = 0.03) in IP1 on stimulation with MSq1 which was not observed by MS438 even at 10 uM. The inset shows the dose dependent increase in IP1 with TSH. The data is plotted after background subtraction. **(B)** Total lysates of FRTL5 cells treated with MS438 10 μM, TSH 1,000 μU/mL and MSq1 10 uM for 24 h and the immunoblots probed for pPKC. MSq1 increased pPKC when compared to the unstimulated cells (lane 0). The 42KD β actin was used as the loading control (*P < 0.05, ***P < 0.0001).

Since the *in-silico* modeling confirmed the potential binding of MSq1 to the TSHR TMD, we examined the key downstream signals that are known to be driven by Gq activation. Activation of PLC was assayed by measuring IP1 accumulation, which showed that MSq1 and TSH were capable of significantly increasing IP1 generation (Figure 4A inset). Furthermore, using phospho-specific antibodies against PKC, we observed that MSq1 significantly enhanced pPKC compared to both TSH and MS438 in thyroid (FRTL5) cells (Figure 4B, upper panel). However, no significant enhancement of pERK or pAKT was observed by MSq1 activation (Figure 4B, lower panel). These downstream signaling studies indicated that MSq1 had the ability to activate the two major arms of Gα_q/11_ signaling as shown by NFAT-luciferase activation and enhanced PKC activation.

### Inhibition of TSH Induced Proliferation by G_α*q*/11_ Activation

**Figure 5:**
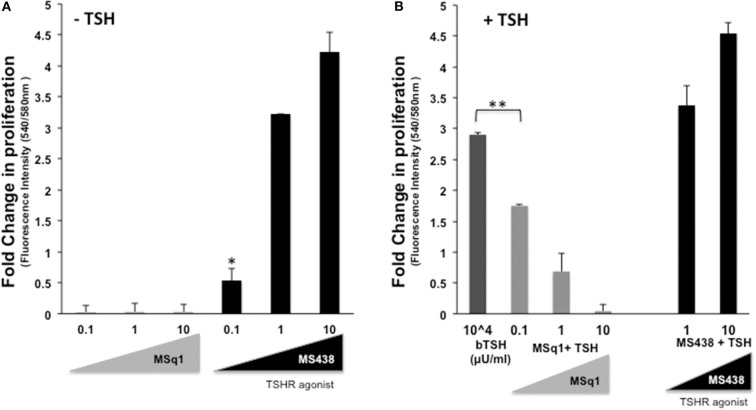
Inhibition of thyrocyte proliferation by MSq1. **(A)** Rat thyrocytes seeded at 30X10^3^/well of 96 well clear bottom black plate and prepared as detailed in materials and method were stimulated with increasing concentrations of MSq1 or MS438. After 48 h of stimulation in basal medium, 1/10th volume of Alamar Blue was added and incubated further at 37°C for 5 h and fluorescence emitted by the dye was measured. As seen here, MSq1 lacked the ability to stimulate proliferation of thyrocytes in comparison to MS438. **(B)** Similar experiments were performed under the same protocol as described above and in the presence of 10^4^μU/mL of TSH. MSq1, indicated by the light gray bars, showed significant dose-dependent inhibition of TSH-induced proliferation. However, this inhibition was specific to MSq1 since MS438, in the presence of TSH, did not inhibit proliferation but rather enhanced TSH induced proliferation at 1 and 10 μM as indicated by black bars (*n* = 3 *P < 0.05, **P < 0.001).

**Figure 6:**
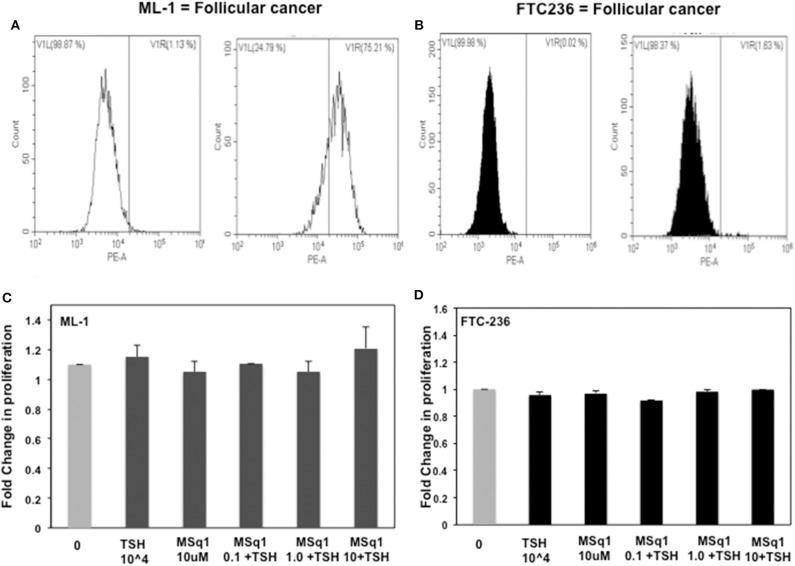
Lack of human thyrocyte proliferation: To ascertain if this inhibition was specific to TSH induced proliferation, we further measured proliferation in two human thyroid follicular carcinoma cell lines with TSH and MSq1. ML-1 cells which are not TSH dependent and express TSHR **(A)** did not show any induction nor inhibition of proliferation **(C)** and likewise FTC236 which lacks TSHR **(B)** also showed no effects on treating with MSq1 **(D)**. These data indicated that the inhibition by MSq1 is observed only by TSH dependent thyroid cells and is not due to any non-specific activation.

The physiological significance of cAMP signaling by Gα_s_ coupling on thyrocyte growth and proliferation is well-established. Since the effect of Gα_q/11_ on thyroid cell proliferation is not clear we examined the action of MSq1 on proliferation of thyrocytes using rat FRTL5 cells. As indicated in Figure 5A, MSq1 failed to enhance basal proliferation of thyrocytes while one of our previously published TSHR agonists (MS438) showed a dose-dependent increase in proliferation and which is known to activate the cAMP-PKA pathway like TSH. In contrast, in the presence of 10^4^ μU/ml of TSH, MSq1 inhibited the TSH induced proliferation of thyrocytes in a dose-dependent manner suggesting a suppressive action of Gq activation on the proliferative capacity of the TSH induced Gs-cAMP-PKA pathway (Figure 5B). This inhibition was only observed in TSH dependent thyrocytes and ML-1 cells derived from a human follicular carcinoma line with a high expression of TSHRs (75% expression of cell surface TSHR as established by flow cytometry) (Figure 6A) or FTC 236 cells, another follicular carcinoma line which totally lacks cell surface TSHR (Figure 6B), did not respond to MSq1 actions (Figures 6C,D). Examining gene expression for common thyroid differentiation markers such sodium iodide symporter (NIS), thyroglobulin (Tg) and the TSHR by qPCR, we did not find these markers to be upregulated in treated cells, suggesting that MSq1 activation of Gα_q/11_ lacked the ability to affect thyrocyte differentiation markers (Figure S3).

### Release of Inhibitory Effect on Proliferation by PKC Inhibition

**Figure 7:**
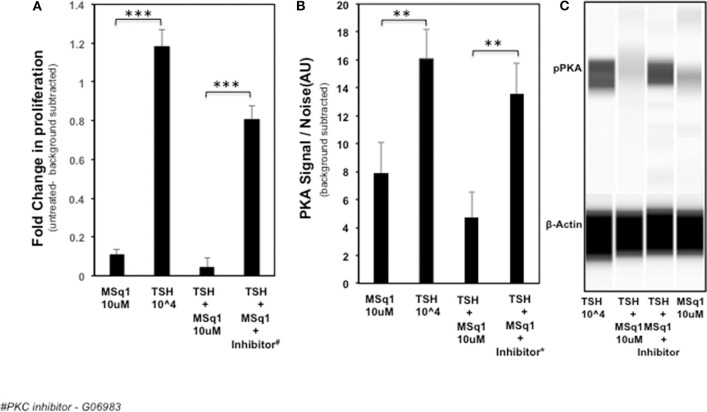
Mechanistic studies on inhibition of thyrocyte proliferation. In order to examine if PKC activation by MSq1 was leading to inhibition of TSH induced proliferation we used a PKC inhibitor (*G06983)*. **(A)** FRTL5 cells were stimulated by MSq1 alone, TSH 10^4^μU/ml alone, TSH +MSq1 and TSH+ MSq1 + PKC inhibitor for 48 h as indicated. MSq1 effectively inhibited TSH induced proliferation but in the presence of this PKC inhibitor (2 uM); proliferation was restored in these cells. Note that the untreated cell data were subtracted as background from all the data (***P < 0.0001). **(B)** Since the possible mechanism of this inhibition was likely to be the result of PKA suppression by the induced PKC, we quantitated the levels of pPKA in the treated samples using immunoblotting in the Wes system. As observed here, PKA was significantly decreased with MSq1 as opposed to TSH alone. This suppression of PKA was released when the cells were treated with TSH and MSq1 in the presence of the PKC inhibitor. The untreated cell data were subtracted as background from all the data (***P < 0.001). **(C)** The lane representations of the WES output are similar to the data that is represented in panel B and shown here with phosphor PKA and β actin bands.

In order to examine the mechanism of the suppression of TSH induced thyroid cell proliferation we used a broad-spectrum PKC inhibitor (G06983) in the presence of TSH and MSq1. As shown earlier, MSq1 treatment at 10 μM caused inhibition of TSH induced proliferation. However, in the presence of the PKC inhibitor, inhibition of proliferation by MSq1 was markedly reduced (Figure 7A). On quantitating the PKA signal using Western blotting with an anti PKA antibody, we observed that cells treated with TSH and MSq1 in the presence of the PKC inhibitor for 48 h showed significantly enhanced PKA signals compared to MSq1 plus TSH or TSH alone (Figures 7B,C). These data demonstrated that enhancement of the PKC signal by MSq1 inhibited the cAMP-PKA pathway induced by TSH activation in the thyrocytes.

## Discussion

TSH is known to induce engagement of all four classes of G protein (2) with the TSHR. However, the major pathway activated by TSH is the Gαs pathway via PKA (29). The consequence of changing this selection is not well-understood. In particular, the role of the Gα_q/11_ pathway via PKC has not been clearly clarified and it is unclear whether overt activation of this pathway has any cellular consequences. Therefore, identifying selective allosteric activators, which are biased to activating a G protein class, is one way of studying the mechanism of TSHR selective signaling and its physiological or pathophysiological effects on thyroid and extra thyroidal TSHRs. This is especially so when knock-out mouse models, which although a very valuable research tool for studying gene function, have their limitations in terms of producing an observable change and may even produce unexpected characteristics which in certain situations cannot be extrapolated to humans (30). In this report, we present data on the identification of a potent Gα_q/11_ activator against the TSHR and our examination of its effects on thyrocytes.

In recent years high-throughput screening assays, combined with *in silico* structural approaches and medicinal manipulations, have resulted in the identification of a number of specific and potent agonists (16, 17, 31) and antagonists (18, 19, 32) against the TSHR which effectively activate or inhibit Gαs initiated signals such as the cAMP-PKA pathway. Using a “tool kit” of CHO-TSHR cells harboring CRE, NFAT, SRF or SRE response elements tagged to luciferase, as shown in Figure 1A, we identified potent and specific Gα_q/11_ selective small molecules. Our search found a molecule (MSq1) unlike our previously reported (17) agonist molecules which is biased toward Gα_q/11_. TSH activates predominantly Gα_s_ (33) and Gα_q/11_ when used in high (non-physiologic) concentrations (34, 35). Coupling of Gα_q/11_ to the TSHR leads to activation of phospholipase C (PLC) which in turn triggers the release of intracellular calcium [Ca^2+^], and NFAT and alternatively activates protein kinase C (PKC) and its downstream effector MAP kinase (MAPK). The normal physiological consequences of activating Gα_s_ in thyrocytes are proliferation, hormone synthesis and thyroglobulin (Tg) iodination (29, 36). However, the physiological or pathophysiological control of Gα_q/11_ signaling in thyrocytes or extrathyroidal TSHRs is not well-characterized despite multiple reports. For example, conditional deletion of Gα_q/11_ in mouse thyroid resulted in hypoplastic thyroid glands and severe hypothyroidism (34). It has also been shown that Gα_q/11_-PKC dependent activation in TSHR transfected papillary cancer cells (line FTC236) resulted in the upregulation of a class of redox and metal ion scavengers which are cysteine-rich proteins known as metallothioneins (MTs) (37). Studies have also shown an indirect relationship of Gα_q/11_ activation to thyroid peroxidase formation (34, 38) and a congenital hypothyroidism phenotype (39).

Our docking studies with a modeled TMD (28) showed that the Gα_q/11_ activator molecule binds within the hydrophobic pocket of the TMD. By this analysis we saw that in addition to overlapping contacts with our agonist MS438 (Gα_s_ dominant), the MSq1 molecule also made contact with some unique resides helping to explain its selective allosteric Gα_q/11_ activation (Table 1). Furthermore, docking MSq1 to the TSH binding surface of the ECD resulted in docking scores that were more than 4 kcal/mol weaker than the top scores observed when docked to the TMD. Such differences represent ~786 times weaker binding indicating that this molecule, like our previously reported small molecules, is can be a allosteric molecule (17).

Despite tremendous progress into the molecular mechanism concerning contacts and activation of G proteins by GPCRs (40, 41) our understanding as to how structurally distinct ligands may lead to the stabilization of different “active states” of the receptors remains open. Homology modeling of the TSHR with the G_q_ heterodimer combined with mutational analysis of the transmembrane domain has indicated the principal determinants leading to the complex interaction (4, 14, 42) suggesting spatial conformation for selective G protein activation.

In this study, we observed that MSq1 is an activator of PLC (Figure 4A) and its downstream effectors—PKC and NFAT activation (Figure 4B). MSq1 showed increased phosphorylation of PKC. However, we failed to see any up regulation in the mRNA levels of thyroid specific genes in contrast to the effect of TSH or our small molecule agonist MS438. On examining the proliferation of these cells, MSq1 alone did not induce any proliferation as seen with MS438 or TSH. It is generally accepted that the proliferation of thyroid cells by TSH is mediated in large part by the cAMP-PKA pathway (43, 44). In contrast, the MSq1 molecule showed the unique ability to suppress TSH induced proliferation. Since we did not observe any blockade of TSH induced cAMP by MSq1 (Figure S4) we hypothesized that suppression must be due to interference with the cAMP-PKA pathway and most likely by PKC activation. There exists cross-talk in downstream signaling of GPCRs (45) and it has been previously shown that PKC can suppress PKA induced activation (46) and functional interference between cAMP/PKA and PKC pathways is possible (47, 48). Thus, experiments carried out in the presence of a PKC inhibitor confirmed that inhibiting PKC in the presence of MSq1 and TSH showed a marked reduction in the suppressive effect of MSq1 on proliferation. Furthermore, pPKA levels showed a significant increase after exposure to the PKC inhibitor. The only study which supports a physiological role for the Gα_q/11_ mediated signaling pathway in TSH induced hormone synthesis (34) was performed in Gα_q/11_ knock out mice. However, the action of MSq1 on proliferation is opposite to the Gα_q/11_ study. Our model would suggest that overt activation of the cAMP-PKA pathway by high concentrations of TSH leading to increased proliferation might be kept in check by the PLC-PKC pathway via Gq and thus maintain a balance in the endogenous proliferative capacity of thyrocytes differing with data that contradicts much of the literature which suggested that TSH stimulates differentiation and not proliferation of normal human thyrocytes (49).

In conclusion, we have identified a novel Gα_q/11_ biased modulator of the TSHR with inhibitory effects on thyrocyte proliferation. The data illustrate the intertwining molecular mechanisms leading to this action. This raises the prospect of modulating biased TSHR signaling for more specific pharmacologic responses.

## Data Availability Statement

The raw data supporting the conclusions of this article will be made available by the authors, without undue reservation, to any qualified researcher.

## Author Contributions

RL responsible for design, execution of the experiments, data analysis, and manuscript writing. SM helped in the experiments with western blots. RM helped in PCR experiments. BT helping in carrying out some facs experiments. MM did the modeling and *in-silco* docking studies. TD helped in data analysis and finalizing of the manuscript.

## Conflict of Interest

TD is a member of the Board of Kronus Inc, Idaho. The remaining authors declare that the research was conducted in the absence of any commercial or financial relationships that could be construed as a potential conflict of interest.

## Abbreviations

TSHR: Thyroid stimulating receptor
GPCR: G protein coupled receptors
GD: Graves’ disease
TMD: transmembrane domain
GAPDH: glyceraldehyde-3-phosphate dehydrogenase.
