# Supplementary figures and images for: A Gq Biased Small Molecule Active at the TSH Receptor
Source: Front Endocrinol (Lausanne). 2020 Jun 26;11:372. doi: 10.3389/fendo.2020.00372 (PMC7333667; doi:10.3389/fendo.2020.00372)

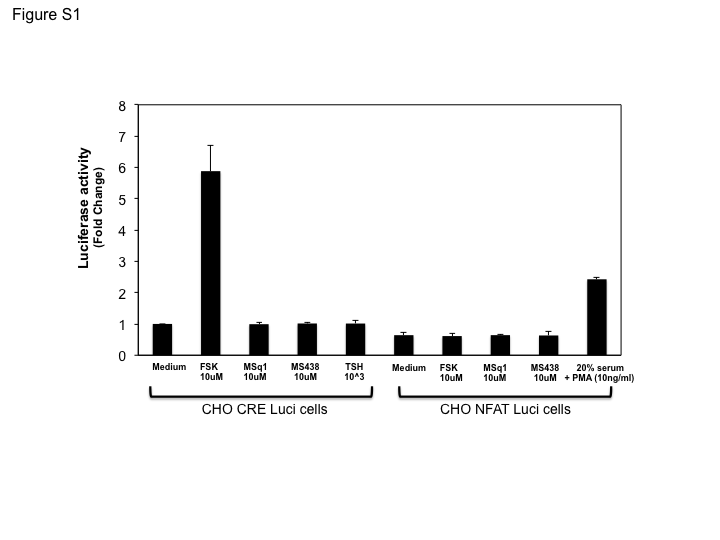

Supplement: Supplementary file 1 [file Image_1.TIFF]

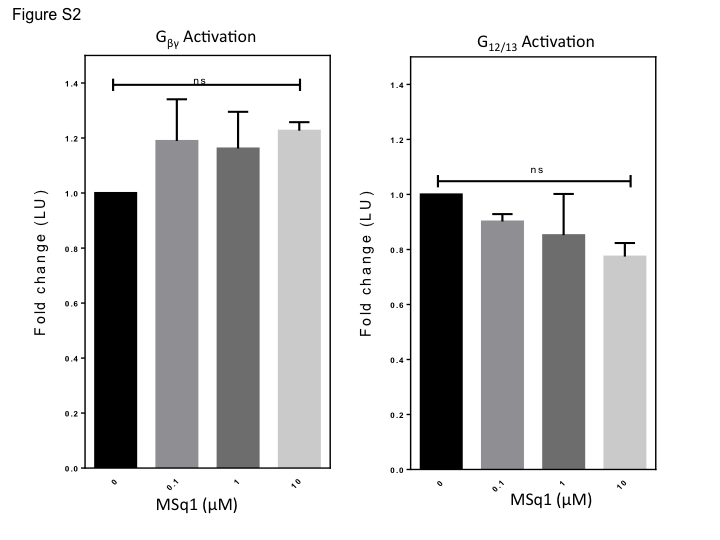

Supplement: Supplementary file 2 [file Image_2.TIFF]

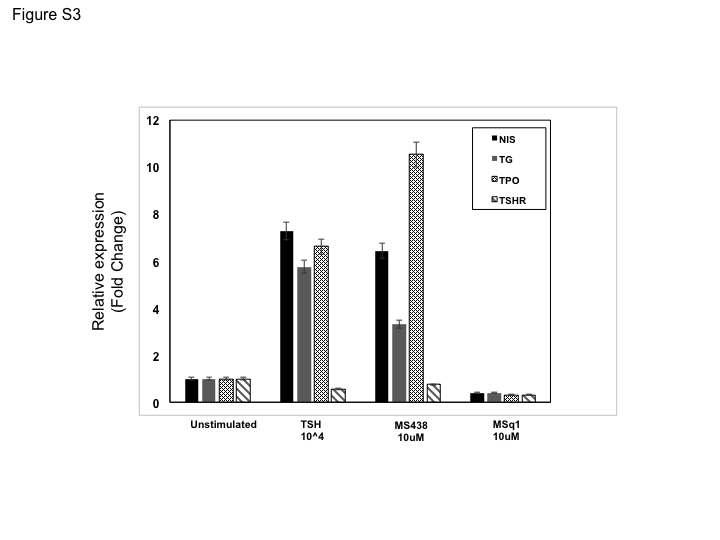

Supplement: Supplementary file 3 [file Image_3.TIFF]

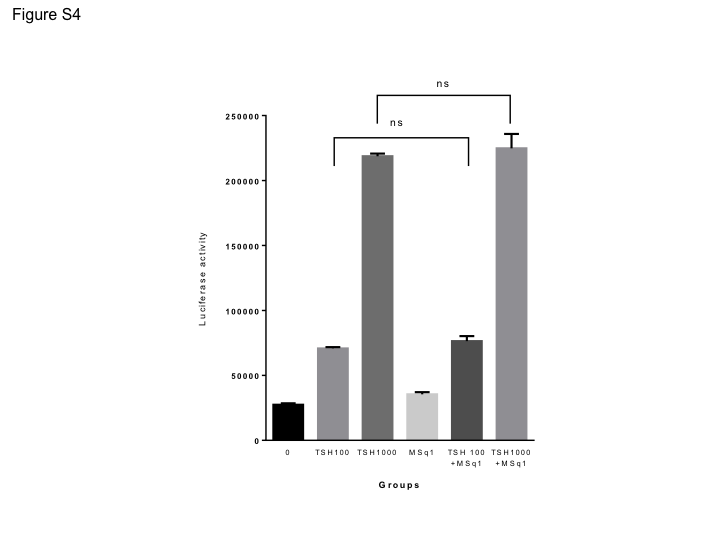

Supplement: Supplementary file 4 [file Image_4.TIFF]
