# Supplementary material for: A Gq Biased Small Molecule Active at the TSH Receptor
Source: Front Endocrinol (Lausanne). 2020 Jun 26;11:372. doi: 10.3389/fendo.2020.00372 (PMC7333667; doi:10.3389/fendo.2020.00372)
Supplement: Supplementary file 5 [file Data_Sheet_1.docx]

**Supplementary Figure Legends**

**FigureS1:** To test specificity of our biased Msq1 molecule we transiently transfected CHO cells with CRE or NFAT luciferase constructs. 48hrs post-transfection the cells were seeded into 96 well plates at 50,000cells/well. After overnight incubation, the cells were then stimulated in triplicates using various test samples as indicated. Msq1 did not activate Gs or Gq in these cells suggesting its specificity to TSHR. The positive controls are indicated by their response to the respective cells (n=2)

**Figure S2 :** To test Msq1 molecule for activation of G_βγ_ and G_12/13_  we used the SRE and SRF luciferase system described in Figure 1. CHOTSHR with these response elements were stimulated in triplicates using various concentrations of MSq1 as indicated. There was no significant (ns) activation of these cells with MSq1 suggesting MSq1 to be specific G_q_  activation (n=2).

**Figure S3:** Quantitative PCR was carried out on cDNA preparations from FRTL5 cells stimulated with various test samples as indicated here and detailed in materials and method. As shown here MSq1 at 10uM lacked the ability to turn on gene expression of any of the thyroid specific markers in contrast to robust increase of thyroid specific markers by MS438 and TSH.

**FigureS4:** To test blockade of cAMP by MSq1 we used our stable CHOTSHR-CRE cells. 50,000cells/well were seeded into 96 well plates and incubated overnight, the cells in triplicate were then pretreated for 30 minutes with the highest concentration of MSq1 (10µM) and then stimulated with two doses of TSH (100 & 1000µU) for 4hrs and the assay was read as previously described. MSq1and TSH alone (n=2).
